# Multi-target effects of bergapten in a vitro periodontitis-mimicking model: suppressing inflammation and oxidative stress while promoting osteogenesis

**DOI:** 10.3389/fphar.2026.1971362

**Published:** 2026-10-29

**Authors:** Sicheng Deng, Hongxia Wei, Yunxin Yang, Xiaofeng Tan, Ruofei Huang, Shaoyong Chen, Haiyan Xue, Yishan Zhang, Fanghong Liu, Rongmin Qiu

**Affiliations:** 1 Key Laboratory of Research and Application of Stomatological Equipment, Education Department of Guangxi Zhuang Autonomous Region, Guangxi Health Commission Key Laboratory of Prevention and Treatment for Oral Infectious Diseases, Guangxi Key Laboratory of Oral and Maxillofacial Surgery Disease Treatment, Guangxi Key Laboratory of Oral and Maxillofacial Rehabilitation and Reconstruction, College & Hospital of Stomatology, Guangxi Clinical Research Center for Craniofacial Deformity, Guangxi Medical University, Nanning, Guangxi, China; 2 Department of Stomatology, Liuzhou Workers’ Hospital, Liuzhou, Guangxi, China

**Keywords:** bergapten, macrophage polarization, natural furanocoumarin, network analysis, oxidative stress, periodontitis-mimicking microenvironment, PI3K/Akt pathway

## Abstract

**Background:**

Bergapten (BG) is a natural furanocoumarin found in various botanical drugs including *Citrus bergamia*, *Angelica archangelica*, and *Citrus medica*, and it’s a bioactive constituent of *Peucedanum guangxiense*, an indigenous species in Guangxi, China. Although BG exhibits anti-inflammatory and osteoprotective properties, its effects relevant to periodontitis-mimicking microenvironment remains unexplored.

**Methods:**

This study integrated network analysis with *in vitro* experimental validation to investigate the multi-target effects of BG in a *Porphyromonas gingivalis* lipopolysaccharide (P.g-LPS) -induced periodontitis-mimicking in vitro model.

**Results:**

Network analysis and molecular docking identified the PI3K/Akt pathway as a candidate pathway, along with predicted binding to CAT, BMP-2, TNF-α, IL-6 and IL-1β. BG at concentration of 5–30 μM did not reduce cell viability in human periodontal ligament stem cells (h-PDLSCs) or RAW264 .7 macrophages under the tested conditions. BG at concentrations of 7.5–30 μM directly promoted osteogenic differentiation of h-PDLSCs beyond baseline levels in the normal-mimicking microenvironment, as evidenced by increased ALP activity, mineralization, and expression of osteogenic markers (*RUNX2, COL-I, OCN*, and *OPN*). Under inflammatory conditions, BG reduced intracellular oxidative activity across concentrations of 7.5–30 μM, suppressed pro-inflammatory cytokines (TNF-α, IL-1β, IL-6), down-regulated M1 macrophages polarization associated markers (*iNOS, CD86*) and NF-κB components (IκBα, p65), while up-regulating anti-inflammatory cytokines (IL-4, IL-10), M2 macrophages polarization-associated markers (Arg-1, CD206) and antioxidant genes (*HO-1,CAT, SOD*). Moreover, BG-treated macrophages conditioned medium partially rescued the osteogenic capacity of h-PDLSCs in inflammation condition, though not to the level of the normal control. The effects might associate with increased phosphorylation levels of PI3K and Akt, and with decreased mRNA expression of NF-κB pathway components.

**Conclusion:**

Collectively, BG was associated with multi-modal effects relevant to periodontitis-mimicking microenvironment, including alleviating oxidative stress, suppressing NF-κB driven inflammation, promoting PI3K/Akt associated osteogenesis, and shifting macrophage polarization-associated markers toward an M2-like profile. Our *in vitro* findings suggest that BG possess multi-modal activities relevant to periodontitis-mimicking microenvironment, warranting further *in vivo* investigation to assess its pharmacological relevance.

## 1 Introduction

Periodontitis is a chronic, multifactorial inflammatory disease initiated by a dysbiotic subgingival microbiome and characterized by a destructive triad of persistent local inflammation, unabated oxidative stress, and progressive alveolar bone loss, ultimately leading to tooth loss (Wong et al., 2026). Epidemiological data from China underscore its significant burden, with reported prevalence rates of 52.8% in adults aged 35–44, 69.3% in those aged 55–64, and 64.6% in individuals aged 65–74 (Jiao et al., 2021). Current clinical management strategies - mechanical debridement, surgery and adjunctive pharmacotherapy–are hampered by anatomical inaccessibility, transient bioactivity, and, most critically, an inability to simultaneously resolve inflammation, counteract oxidative stress, and promote bone regeneration (Sun et al., 2026; Wang et al., 2026; Kim et al., 2021; Duarte et al., 2025). The pathogenesis is largely driven by *Porphyromonas gingivalis* (*P. gingivalis*) lipopolysaccharide (LPS), which triggers inflammatory related signaling, excessive release of pro-inflammatory cytokines, reactive oxygen species (ROS) burst, and M1 macrophage polarization (Xiang et al., 2025; Anderson, 2000; Cui et al., 2017; Sculley and Langley-Evans, 2002). This hostile microenvironment not only perpetuates tissue destruction but also severely impairs the osteogenic differentiation of human periodontal ligament stem cells (h-PDLSCs) (Xu et al., 2020). Hence, a therapeutic agent that concurrently tackles all three pathological pillars is urgently needed. Although the *P.g*-LPS-stimulated model is a simplified surrogate for the complex periodontal inflammatory milieu, it is widely accepted as a standard *in vitro* system for studying host-pathogen interactions, as *P. gingivalis* is a keystone pathogen and its LPS activates TLR4 signaling to release proinflammatory cytokines that mimic clinical conditions (Xu et al., 2020; Daup et al., 2006; Tang et al., 2016). This model provides a reproducible platform for initial investigation before more complex *in vivo* validation.

Botanical drugs and their metabolites offer an attractive source of multi-target candidates due to their inherent biocompatibility and low toxicity. Bergapten (BG, 5-methoxypsoralen), a natural furanocoumarin, is widely found in Apiaceae and Rutaceae plants. Notably, BG is a bioactive furanocoumarin widely distributed across diverse plant families, particularly Apiaceae and Rutaceae, which are found in various regions worldwide, including East Asia, the Mediterranean, and South America. Plants containing BG, such as *Citrus bergamia*, *Angelica archangelica*, and *Peucedanum* species–range from East Asian botanical drugs to European folk remedies–for the treatment of inflammatory disords, respiratory ailments, and gastrointestinal complaints (Liang et al., 2021). This long-standing ethnopharmacological history underscores the compound’s established safety and therapeutic potential across cultures. Moreover, BG has been the subject of extensive pharmacological research, demonstrating a broad spectrum of bioactivities that further support its relevance as a multi-target natural product. Recent pharmacological studies have validated some of these uses, showing that BG exhibits antitumor (Sumiyoshi et al., 2014) and hypoglycemic (Fang et al., 2016) effects. Moreover, BG has been shown to enhance osteoblast differentiation, suppressing osteoclastogenesis (Tang et al., 2008; Zheng et al., 2014), and inhibiting NLRP3 inflammasome activation via mitophagy modulation (Luo et al., 2023). Although previous studies have reported anti-inflammatory and osteogenic activities of BG in various contexts, its integrated effects on macrophage polarization, redox balance, and osteogenic differentiation within a single *in vitro* periodontitis-mimicking model have not been systematically evaluated. Moreover, whether BG-treated macrophages exert a paracrine influence on the osteogenic differentiation of h-PDLSCs under inflammatory conditions remains further explored.

To bridge this gap, we employed an integrated approach combining network analysis prediction with *in vitro* experimental validation in a *P. gingivalis* LPS-induced periodontitis-mimicking model. We hypothesize that BG can concurrently reduce intracellular oxidative activity and upregulate antioxidant defenses, suppress inflammation and modulate macrophage polarization-associated markers towards an M2-like profile, and promote h-PDLSCs osteogenic differentiation through related signaling pathways. By providing evidence for the traditional use of BG-containing medicinal plants and highlighting their multi-target therapeutic profile, our findings position BG as a promising natural candidate for periodontal therapy and provide a preliminary framework for discovering complex natural products in inflammatory diseases.

## 2 Materials and methods

### 2.1 Network analysis

Putative targets of BG were retrieved from the Traditional Chinese Medicine Systems Pharmacology Database (TCMSP, https://tcmspe.com/tcmsp.php), the IUPHAR/BPS Guide to Pharmacology (https://www.guidetopharmacology.org/), and the Comparative Toxicogenomics Database (CTD, https://ctdbase.org/). Target genes from multiple sources were compiled and duplicates were removed. Only targets annotated to *Homo sapiens* were retained for subsequent analysis. All target genes were standardized to official gene symbols using UniProt (https://www.uniprot.org/). Periodontitis-related genes were retrieved from the GeneCards database (https://www.genecards.org/) using the keyword “periodontitis”, and gene with a relevance score above the default threshold were included. Overlapping genes between BG target set and the periodontitis-associated gene set were identified via Venn diagram analysis using R software (version 4.2.0), yielding candidate overlapping targets for further analysis.

For functional annotation, the intersecting target genes were converted to Entrez IDs and subjected to Gene Ontology (GO) enrichment and Kyoto Encyclopedia of Genes and Genomes (KEGG) pathway analysis using the clusterProfiler package in R. A significance threshold of *p* ≤ 0.05 and *q* ≤ 0.05 was applied to predict enriched biological processes and candidate signaling pathways.

To computationally assess the potential interactions between BG and the candidate target proteins, molecular docking was performed using the CB-Dock 2 online server (https://cadd.labshare.cn/cb-dock2/). The 3D structure of BG was downloaded from PubChem (CID: 2355) in SDF format. The crystal structures of CAT (PDB:1DGB), BMP-2 (PDB:1ES7), TNF-α (PDB: 2A8H), IL-6 (PDB:1N26), and IL-1β (PDB: 3WO3) were retrieved from the RCSB Protein Data Bank. CB-Dock 2 automatically detected potential binding pockets and performed docking using AutoDock Vina with default parameters. The top-ranked docking pose for each target was selected based on the Vina score. The predicted binding energies, cavity volumes, center coordinates, docking box sizes, and contact residues are summarized in Table 2. All docking results are computational predictions, and the limitations of docking as a non-experimental tool are acknowledged. *In vitro* validation was not performed in this study.

### 2.2 Cell culture and identification

RAW264.7 murine macrophages were obtained from Hycyte Biotechnology Co., Ltd (China) and cultured per the manufacturer’s protocol. The medium was renewed every 1–2 days, and cells were passaged upon reaching 80%–90% confluence to maintain exponential growth.

Human periodontal ligament stem cells were isolated from clinically healthy premolars or third molars extracted from six independent donors (aged 16–25 years; 3 males and 3 females) at the Stomatological Hospital of Guangxi Medical University, under an approved ethical protocol (No, 20200050). Informed consents were obtained from all donors or their legal guardians. Periodontal tissues were rinsed with Phosphate-buffered saline (PBS; Gibco, USA) containing 1% penicillin/streptomycin (Gibco, USA), then minced into 1–3 mm^2^ explants and digested with 3 mg/mL type I collagenase (Sigma, USA) at 37 °C for 30 min. The digests were centrifugated at 1200rpm for 5 min, and the pellet was resuspended in α-minimum essential medium (α-MEM; Gibco, USA) supplemented with 1% penicillin/streptomycin and 10% fetal bovine serum (FBS; Gibco, Grand Island, USA). Cells were maintained at 37 °C in a 5%CO_2_ humidified atmosphere, with medium changed every 3 days. At 80% confluence, cells were passaged using 0.25% trypsin-EDTA (Wisent, Canada). Cells from all donors were pooled at passage 1 to minimize donor-to-donor variability and were used at passage 3 for all experiments.

For immunophenotype characterization, h-PDLSCs were harvested, washed, and resuspended in PBS at 1 × 10^6^ cells/ml. Aliquots (50 µL) were incubated with 5 µL each of CD34^−^fluorescein isothiocyanate (FITC), CD90^−^allophycocyanin (APC), CD146-peridinin-chlorophyll-protein complex-cyanine 5.5 (PerCP-Cy5.5), and STOR-1- phycoerythrin (PE) antibodies (Abcam, UK) for 20 min in the dark. For flow cytometric immunophenotyping, the gating strategy was set based on forward scatter (FSC) and side scatter (SSC) to exclude debris and doublets, with at least 10,000 events acquired per sample. Fluorescence was analyzed on a BD flow cytometer (BD Bio-science, USA).

Multilineage differentiation capacity was accessed by osteogenic and adipogenic induction. Cells were seeded in 6-well plates at 2 × 10^4^ cells/well. For osteogenesis, cells at 60%–70% confluence were cultured in osteogenic induction medium (OIM; Cyagen, USA) for 21 days. For adipogenesis, over-confluent cells were maintained in adipogenic induction medium (AIM; Cyagen, USA) for 3–4 weeks, with medium changed as recommended. After induction, cells were fixed with 4% paraformaldehyde (Solarbio, China) for 30 min and stained with Alizarin Red S (ARS) or Oil Red O (Beyotime, China) to visualize mineralized nodules and lipid droplets, respectively.

### 2.3 Cell counting Kit-8 (CCK-8) assay

Cell viability was evaluated using the CCK-8 assay to determine the cytotoxicity prolife of BG. RAW264.7 cells and h-PDLSCs were seeded in 96-well plates at 5 × 10^3^ cells/well. h-PDLSCs were treated with BG at concentrations of 5, 7.5, 15, 30, 60 μM (Aladdin®, China) for 24, 48, and 72h, while RAW 264.7 cells were exposed to the same concentration range for 24 h only. At each designated time point, 10 µL of CCK-8 reagent (Solarbio, China) was added to each well, and plates were incubated at 37 °C in a 5%CO_2_ humidified atmosphere in the dark for 2 h. Absorbance was recorded at 450 nm using a microplate reader (TECAN Infinite M200Pro, canton of Zürich, Switzerland). Cell viability was calculated as:

$$\text{Cell viability}\left(\%\right)=\left({\text{OD}}_{\text{experimental}\;\text{group}}-{\text{OD}}_{\text{blank}}\right)/\left({\text{OD}}_{\text{control}\;\text{group}}-{\text{OD}}_{\text{blank}}\right)\times 100\%$$

To access the effect of BG long-term proliferation, h-PDLSCs were seeded at 2 × 10^3^ cells/well in 96-well plates and exposed to the same BG concentration gradient. On days 0, 1, 3, 5, and 7, CCK-8 reagent was added and incubated as described above, and absorbance was measured to generate a growth curve reflecting proliferation dynamics under BG treatment.

### 2.4 Reactive oxygen species staining

To evaluate the antioxidative capacity of BG, intracellular ROS levels were measured in RAW264.7 cells using the fluorescent probe 2′,7′-dichlorodihydrofluorescein diacetate (DCFH-DA). Cells were seeded in 48-well plates at 2 × 10^4^ cells/well and allowed to adhere for 24 h. To establish an inflammatory and oxidative stress environment, cells in the inflammation and experimental groups were pretreated with 1 μg/ml *P.g-*LPS (Sigma, USA) for 2h, followed by exposed to BG at concentrations of 5–30 μM for 6 h. The normal control and inflammation groups received vehicle only. After treatment, cells were incubated with 10 μM DCFH-DA in serum-free medium at 37 °C for 30 min in the dark. Cells were then washed three times with PBS, and ROS-associated green fluorescence was visualized and photographed under a fluorescence microscope (Thermo Fisher Scientific, USA).

### 2.5 Enzyme linked immunosorbent assay (ELISA)

To gauge the anti-inflammatory effect of BG, cytokine secretion by RAW264.7 cells was quantified using ELISA Cells were seeded in 6-well plates at 2 x 10^5^ cells/well and subjected to the same inflammatory induction protocol as described for the CCK-8 assay. Briefly, after pretreatment with 1 μg/ml *P.g*-LPS for 2h, experimental groups were treated with 5–30 μM BG, while normal and inflammation groups received vehicle only. Supernatants were collected after 24 h and centrifugated at 1000 rpm for 5 min to remove debris. Levels of pro-inflammatory cytokines (IL-1β, IL-6, TNF-α), and anti-inflammatory cytokines (IL-4, IL-10) were measured using commercial ELISA kits (MEIMIAN, China) strictly according to the manufacturer’s protocols.

### 2.6 Alkaline phosphatase (ALP) staining and activity analysis

To evaluate the early osteogenic effects of BG, h-PDLSCs were seeded in 48-well plates at 2 × 10^4^ cells/well. Upon reaching 70%–80% confluence, cells were divided into the following groups: Normal group (OIM only), Inflammation group (OIM+1 μg/ml *P.g* LPS), and experimental groups (OIM+1 μg/ml *P.g* LPS+5, 7.5, 15, or 30 μM BG). After 7 days of osteogenic induction, cells were fixed with 4% paraformaldehyde for 10 min and stained using a BCIP/NBT Alkaline Phosphatase Color Development Kit (Beyotime, China) to visualize ALP activity. For quantification, culture supernatants were collected and centrifugated at 1200 rpm for 10 min, and ALP activity was measured by Alkaline Phosphatase Assay Kit (Nanjing Jiancheng Bioengineering Institute, China) following the manufacturer’s instructions.

### 2.7 Alizarin Red S (ARS) staining and semi-quantitative analysis

To assess the late-stage osteogenic potential of BG, matrix mineralization was evaluated after 21 days of osteogenic induction under the same grouping and treatment regimens as described for the ALP assay. Following induction, h-PDLSCs were fixed with 4% paraformaldehyde and stained with ARS solution for 5 min, then gently rinsed with distilled water to remove non-specific staining. For semi-quantitation, the stained mineralized nodules were solubilized in 10% cetylpyridinium chloride (CPC; Aladdin®, China), and the absorbance of the extracted dye was measured at 562 nm using a microplate reader.

### 2.8 Real-time quantitative polymerase chain reaction (RT-qPCR)

To systemically assess the osteogenic effect of BG on h-PDLSCs and its regulatory roles in macrophage polarization, antioxidant defence, and nuclear Factor kappa-B (NF-κB) pathway activity in RAW264.7 cells, RT-qPCR was performed. h-PDLSCs were treated with BG at the same concentration gradient as described in the ALP analysis. RAW264.7 cells were divided into three groups: normal group, inflammatory group (*P.g*-LPS induced only), and experimental groups (*P.g* LPS induced +7.5, 15, or 30 μM BG). Total RNA was extracted using TRIzol reagent (Thermo Fisher, USA) and reverse-transcribed into complementary DNA by Hiscript III qRT SuperMix (Vazyme, China). Quantitative PCR was then performed with ChamQ SYBR qPCR Master Mix (Vazyme, China) on a QuantStudio5 Real-Time PCR System (Thermo Fisher, USA). Relative mRNA expression levels were calculated using the 2^−ΔΔCt^ method, with GAPDH serving as the internal reference gene for h-PDLSCs and β-actin for RAW264.7 cells. The stability of these reference genes under the experimental conditions was verified by preliminary RT-qPCR (Ct values showed <1 cycle variation across group).

Primer sequences are listed in Table 1.

**TABLE 1:** Primer sequences in RT-qPCR.

Name	Forward (5′-3′)	Reverse (3′-5′)
*iNOS*	*5′-ACT​CAG​CCA​AGC​CCT​CA-3′*	*5′-CTC​TGC​CTA​TCC​GTC​TCG​T-3′*
*CD86*	*5′-AGC​ACT​ATT​TGG​GCA​CAG​AGA​AAC-3′*	*5′-GTG​AAG​TAG​AGT​CCA​GTT​GTT​C-3′*
*Arg-1*	*5′-AGG​AGA​TGC​AAA​GGA​GGA​A-3′*	*5′-CCT​ACC​ACA​CCA​AAA​GCC-3′*
*CD206*	*5′-TGG​CAA​GTA​TCC​ACA​GCA-3′*	*5′-GGT​TCC​ATC​ACT​CCA​CTC​A-3′*
*HO-1*	*5′-TTA​CCT​TCC​CGA​ACA​TCG​AC-3′*	*5′-TCC​TCT​GTC​AGC​ATC​ACC​TG-3′*
*SOD*	*5′-CTG​GAG​CCA​CAC​ATT​AAC​GC-3′*	*5′-CGG​TGG​CGT​TGA​GAT​TGT​TC-3′*
*CAT*	*5′-AGA​GGA​AAC​GCC​TGT​GTG​AG-3′*	*5′-GCG​TGT​AGG​TGT​GAA​TTG​CG-3′*
*IκBα*	*5′-GCC​AGG​AAT​TGC​TGA​GGC​ACT​T-3′*	*5′-GTC​TGC​GTC​AAG​ACT​GCT​ACA​C-3′*
*P65*	*5′-TCC​TGT​TCG​AGT​CTC​CAT​GCA​G-3′*	*5′-GGT​CTC​ATA​GGT​CCT​TTT​GCG​C-3′*
*TNF-*α	*5′-GGT​GCC​TAT​GTC​TCA​GCC​TCT​T-3′*	*5′-GCC​ATA​GAA​CTG​ATG​AGA​GGG​AG-3′*
*IL-6*	*5′-TAC​CAC​TTC​ACA​AGT​CGG​AGG​C-3′*	*5′-CTG​CAA​GTG​CAT​CAT​CGT​TGT​TC-3′*
*β-actin*	*5′-ATG​GTG​GGA​ATG​GGT​CAG​AA-3′*	*5′-TCT​CCA​TGT​CGT​CCC​AGT​TG-3′*
*ALP*	*5′-GCT​GTG​GAA​GGC​TCT​GGA​AAG​TC-3′*	*5′-CAG​TCA​CTC​TGG​CAC​TCA​GGT​TTC-3′*
*RUNX2*	*5′-CCA​CTG​AAC​CAA​AAA​GAA​ATC​CC-3′*	*5′-GAA​AAC​AAC​ACA​TAG​CCA​AAC​GC-3′*
*COL-1*	*5′-TGA​TCG​TGG​TGA​GAC​TGG​TCC​TG-3′*	*5′-CTT​TAT​GCC​TCT​GTC​GCC​CTG​TTC-3′*
*OCN*	*5′-AGG​GCA​GCG​AGG​TAG​TGA​AGA​G-3′*	*5′-GGT​CAG​CCA​ACT​CGT​CAC​AGT​C-3′*
*OPN*	*5′-CAG​CCG​TGG​GAA​GGA​CAG​TTA​TG-3′*	*5′-TCA​CAT​CGG​AAT​GCT​CAT​TGC​TCT​C-3′*
*GAPDH*	*5′-TCA​TGG​GTG​TGA​ACC​ATG​AGA​A -3′*	*5′-GGC​ATG​GAC​TGT​GGT​CAT​GAG-3′*

### 2.9 Western blot (WB)

To assess the network analysis prediction that the PI3K/Akt signaling pathway is a key target of BG, WB was performed to assess protein expression levels. h-PDLSCs were treated following the same grouping as described for RT-qPCR. Total protein was extracted using RIPA lysis buffer (Beyotime, China) and quantified with a BCA Protein Assay Kit (Beyotime, China). Equal amounts of protein samples were separated by 12.5% sodium dodecyl sulfate polyacrylamide gel electrophoresis (SDS-PAGE) Gel (Epizyme, China) and transferred onto polyvinylidene fluoride (PVDF) membranes (Servicebio, China). After blocking with 5% (w/v) skimmed milk for 1 h at room temperature, membranes were incubated overnight at 4 °C with primary antibodies against Akt, p-Akt, PI3K, p-PI3K, and β-tubulin (ZEN-Bio, China, 1:1000 dilution). Following washing with Tris-buffed saline containing 0.1% Tween-20 (TBST, Solarbio, China), membranes were incubated with horseradish peroxidase (HRP)-conjugated secondary antibodies (ZEN-Bio, China, 1:10000 dilution) for 1 h at room temperature. Protein bands were visualized using enhanced chemiluminescence (ECL) reagents (SEVEN, China) and imaged with a ChemiDoc Touch System (BIO-RAD, USA). Band intensities were quantified using ImageJ software, and the ratios of phosphorylated to total protein were calculated for statistical analysis.

### 2.10 Osteoimmunomodulation analysis

To evaluate whether BG-treated macrophages could influence h-PDLSCs osteogenic differentiation through paracrine factors, conditioned medium (CM) was collected from RAW 264.7 cells. Cells were seeded in 10 cm culture dishes at 2 × 10^5^ cells/dish and treated for 24 h under the following conditions: normal control, inflammation group (*P.g*-LPS stimulated only), and experimental group (*P.g*-LPS stimulated +7.5, 15, or 30 μM BG). After incubation, CM from each group was collected, centrifuged to remove cellular debris, filtered through 0.22 μm pore-size membranes, and stored at −80 °C for subsequent use.

h-PDLSCs were planted in 6-well plates at 2 × 10^4^ cells/well. Upon reaching 70%–80% confluence, cells were cultured in a 1:1 mixture of standard OIM and the corresponding CM, with the groups assigned according to the CM source. After 7 days of culture, ALP staining and activity were assessed, and total RNA was extracted for RT-qPCR analysis of osteogenic marker genes. After 21 days, matrix mineralization was evaluated by ARS staining and semi-quantification.

### 2.11 Statistical analysis

All experiments were performed with three independent biological replicates. Within each biological replicate, three technical replicates were included for experiments. Statistical analysis was performed using SPSS 25.0 (IBM, USA) and GraphPad Prism 10 (GraphPad Software, USA). Data are presented as mean ± standard deviation (SD) of the three biological replicates. For multiple-group comparisons, one-way ANOVA was used when homogeneity of variance was confirmed by Levene’s test, followed by Tukey’s *post hoc* test for pairwise comparisons. When homogeneity of variance was violated, Welch’s ANOVA with Games-Howell *post hoc* test was applied. Significance levels are indicated as *p <0.05, **p <0.01, ***p <0.001.

## 3 Result

### 3.1 Network analysis

Network analysis identified 39 potential targets of BG and 95 periodontitis-related genes, with an intersection of 5 overlapping candidate genes (Figure 1A). A protein-protein interaction (PPI) network constructed from these targets comprised 5 nodes and 15 edges, indicating a tightly interconnected functional module (Figure 1B).

**FIGURE 1:**
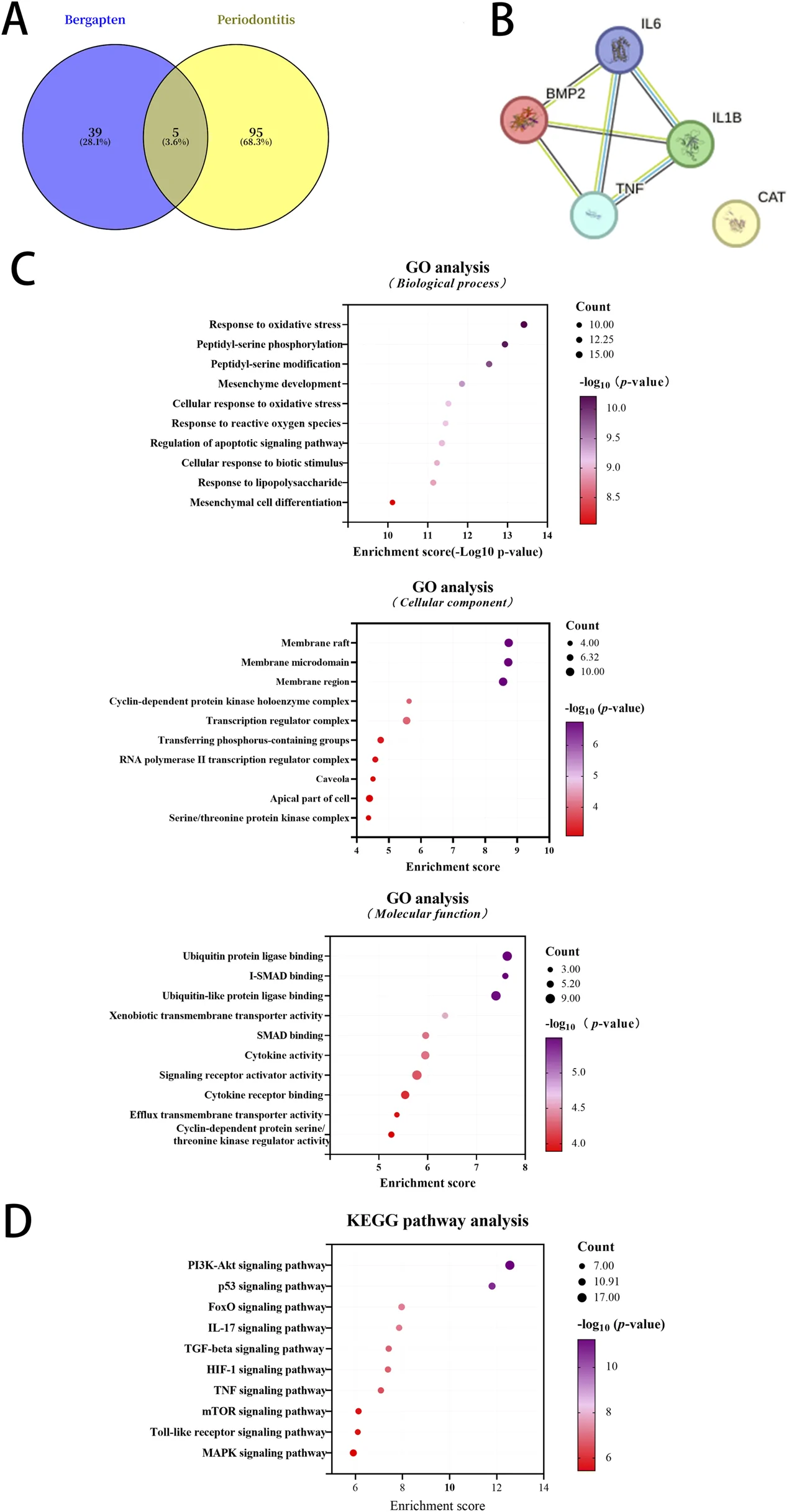
Network analysis. **(A)** Venn diagram. **(B)** protein interaction networks. **(C)** GO analysis. **(D)** KEGG analysis.

GO enrichment analysis revealed that the top enriched biological processes were closely associated with oxidative stress response, peptidyl-serine phosphorylation, and mesenchyme development. Key cellular components included membrane rafts and transcription regulator complex, while major molecular functions involved ubiquitin protein ligase binding and SMAD binding (Figure 1C). KEGG pathway analysis identified 163 significantly enriched pathways (*p*<0.05), among which the top five were PI3K/Akt, p53, FoxO, IL-17, TGF-β signaling pathways (Figure 1D). Collectively, these exploratory results provide preliminary clues that BG may exert its effects through multimodal regulation of signaling networks, with the PI3K/Akt pathway emerging as a hypothesis-generating clue for subsequent experiments. All enrichment results were corrected for multiple comparisons using the Benjamini–Hochberg method, and the full lists of enriched GO terms and KEGG pathways with their respective *p*-adjust and *q*-values are provided in the supplementary materials.

The two-dimensional and three-dimensional structures of BG were retrieved from the PubChem (Figures 2A,B). Molecular docking simulations were performed to predict the binging affinity between BG and the five candidate targets (CAT, BMP-2, TNF-α, IL-6, and IL-1β). The results showed that BG was predicted to bind to all five targets, with binding energies consistently below −5.0 kcal/mol (Table 2; Figures 2C–G), suggesting potential predicted interactions. These *in silico* predictions are exploratory and do not establish direct or stable binding.

**FIGURE 2:**
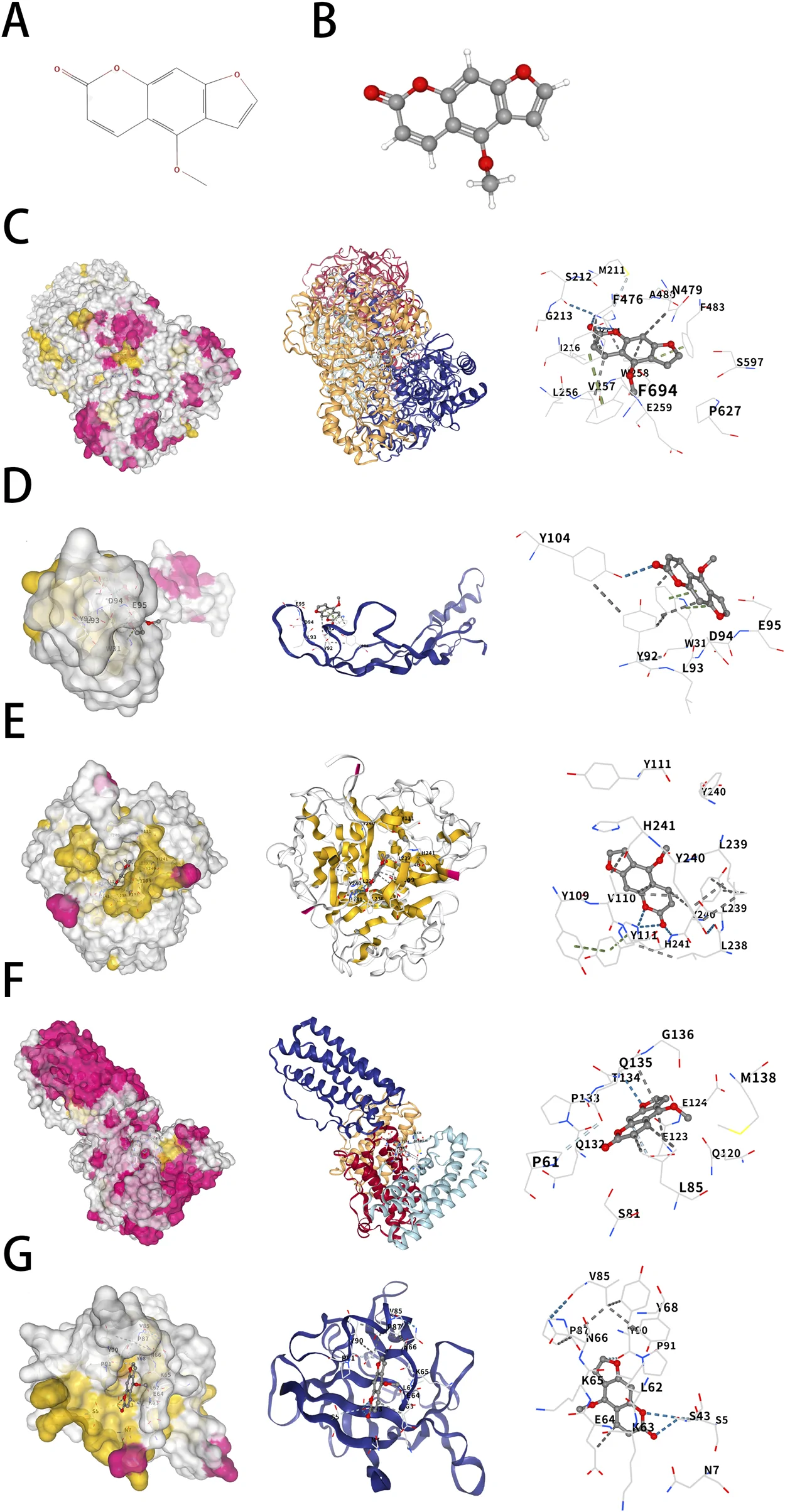
Structure of bergapten and molecular docking. **(A)** 2D structure. **(B)** 3D structure. **(C–G)** Predicted binding poses of BG to CAT **(C)**, BMP-2 **(D)**, TNF-α **(E)**, IL-6 **(F)**, and IL-1β **(G)**.

**TABLE 2:** Predicted binding parameters of bergapten to candidate target proteins.

Protein	PDB ID	Ligands	Predicted binding energy (kcal/mol)	Cavity volume (Å3)	Center (x, y, z)	Docking box size (x, y, z)	Contact residues
*CAT*	*1DGB*	*Bergapten*	*−8.7*	*1199*	*−14.198, 15.373, 30.130*	*18, 18, 18*	*ARG:79:B, ALA:80:B, VAL:81:B, HIS:82:B, ARG:119:B, PHE:120:B, SER:121:B, GLY:138:B, PHE:139:B, ALA:140:B, VAL:153:B, GLY:154:B, ASN:155:B, PHE:160:B, ALA:165:B, ILE:166:B, PHE:168:B, HIS:229:B, VAL:343:B, MET:344:B, PHE:345:B, LEU:361:B, GLY:364:B, ARG:365:B, SER:368:B, TYR:369:B, THR:372:B, GLN:373:B, ARG:376:B, PHE:64:D, ILE:68:D, PHE:71:D, ASP:72:D*
*BMP-2*	*1ES7*	*Bergapten*	*−6.0*	*51*	*−0.602, −36.127, −5.795*	*18, 18, 18*	*VAL:21:A, PHE:23:A, ILE:26:A, TRP:28:A, TRP:31:A, TYR:38:A, ALA:40:A, TYR:41:A, TYR:92:A, LEU:93:A, ASP:94:A, GLU:95:A, TYR:104:A, ASP:106:A, MET:107:A, VAL:108:A, VAL:109:A*
*TNF-a*	*2A8H*	*Bergapten*	*−7.0*	*833*	*−41.366, 104.653, −18.769*	*18, 18, 27*	*TYR:109:A, VAL:110:A, TYR:111:A, LEU:238:A, LEU:239:A, TYR:240:A, HIS:241:A, ALA:108:B, TYR:109:B, VAL:110:B, TYR:111:B, LEU:238:B, LEU:239:B, TYR:240:B, HIS:241:B, TYR:109:C, VAL:110:C, TYR:111:C, LEU:238:C, LEU:239:C, TYR:240:C, HIS:241:C*
*IL-6*	*1N26*	*Bergapten*	*−7.1*	*582*	*7.246, 134.393, −18.120*	*18, 18, 18*	*PRO:58:A, PRO:61:A, SER:81:A, PHE:84:A, LEU:85:A, GLY:88:A, GLN:132:A, PRO:133:A, THR:134:A, GLN:135:A, GLY:136:A, ALA:137:A, MET:138:A, THR:116:C, TRP:119:C, GLN:120:C, GLU:123:C, GLU:124:C*
*IL-1*β	*3WO3*	*Bergapten*	*−5.9*	*199*	*12.939, −18.591, −14.145*	*18, 18, 18*	*VAL:3:A, ARG:4:A, SER:5:A, LEU:6:A, ASN:7:A, SER:43:A, SER:45:A, GLY:61:A, LEU:62:A, LYS:63:A, GLU:64:A, LYS:65:A, ASN:66:A, LEU:67:A, TYR:68:A, VAL:85:A, PRO:87:A, TYR:90:A, PRO:91:A, SER:153:A*

Docking was performed using the CB-Dock2 online server with AutoDock Vina. for each target, the top-ranked binding pose with the lowest Vina score was selected. The center coordinates and docking box sizes are given in Å. Contact residues are listed with chain identifiers. All values are computational predictions and do not represent experimentally determined binding affinities.

### 3.2 Isolation, culture, and characterization of cells

Primary h-PDLSCs were successfully isolated via the tissue block culture method. Cell outgrowth from the periphery of tissue was observed within 7–14 days (Figure 3A). Following purification by limited dilution, the cells displayed a homogeneous, spindle-shaped fibroblast-like morphology (Figure 3B). Colony-forming unit assays support their self-renewal capacity, as evidenced by the formation of discrete fibroblastic colonies (Figures 3C,D).

**FIGURE 3:**
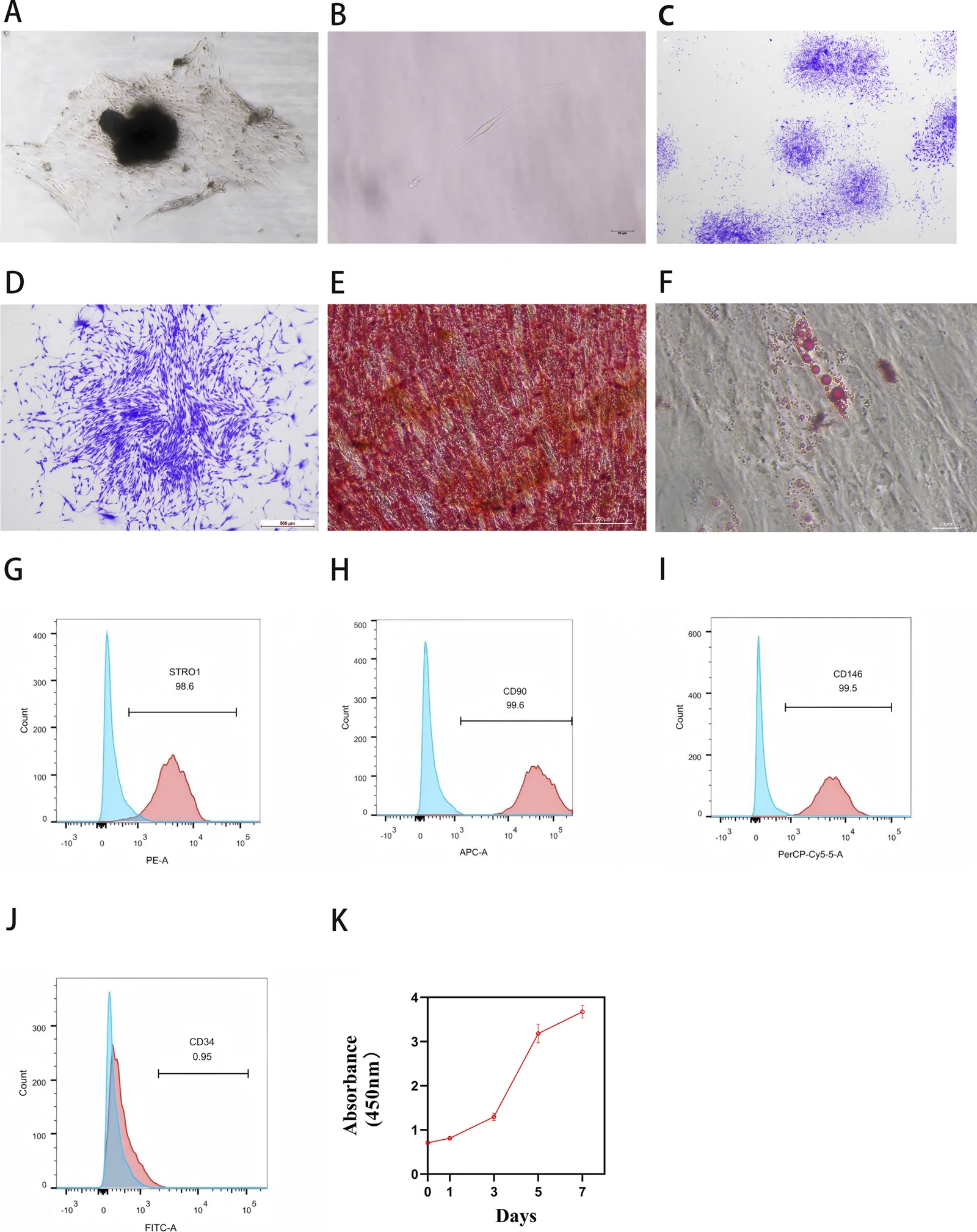
Isolation, culture, and characterization of human periodontal stem cells. **(A)** Tissue block culture method (1×). **(B)** Single cell of h-PDLSCs (20×). **(C)** Clone units (1×). **(D)** Single clone unit (4×). **(E)** Osteogenic differentiation (4×). **(F)** Adipogenic differentiation (10×). **(G–J)** The MSCs phenotypic molecular surface markers of h-PDLSCs. **(K)** Growth curve of h-PDLSCs.

Under osteogenic induction, ARS staining revealed distinct mineralized nodules formation after 21 days (Figure 3E). Similarly, adipogenic induction led to intracellular lipid droplets accumulation, as visualized by Oil Red O staining (Figure 3F), indicating the multipotent differentiation potential of isolated cells. Flow cytometric immunophenotyping demonstrated that the cells were highly positive for mesenchymal stem cell markers CD90 (99.6%), CD146 (99.5%) and STRO-1 (98.6%), while negative for the hematopoietic marker CD34 (0.95%) (Figures 3G–J). Proliferation kinetics analysis showed a characteristic growth patten comprising an initial lag phase (days 0–3), a logarithmic growth phase (days 3–5), and a final plateau phase (days 5–7) (Figure 3K). Collectively, these results suggest that the isolated cells fulfill the minimal criteria for multipotent mesenchymal stem cells and, based on their tissue origin, can be identified as periodontal ligament stem cells, supporting their suitability for subsequent mechanistic studies.

### 3.3 Cytotoxicity analysis

The cytocompatibility of BG was evaluated in both h-PDLSCs and RAW264.7 macrophages. In h-PDLSCs, short-term exposure (24–72 h) to BG at concentrations up to 30 μM did no significantly affect cell viability compared with the untreated control (*p* > 0.05). In contrast, treatment with 60 μM BG induced a marked reduction in viability at all time points (*p* < 0.05), indicating a concentration-dependent cytotoxic threshold (Figures 4A–C). Long-term proliferation analysis further indicated that BG at 5–30 μM did not alter the growth kinetics of h-PDLSCs, whereas 60 μM BG significantly suppressed cell proliferation from day 3 onward (Figure 4D).

**FIGURE 4:**
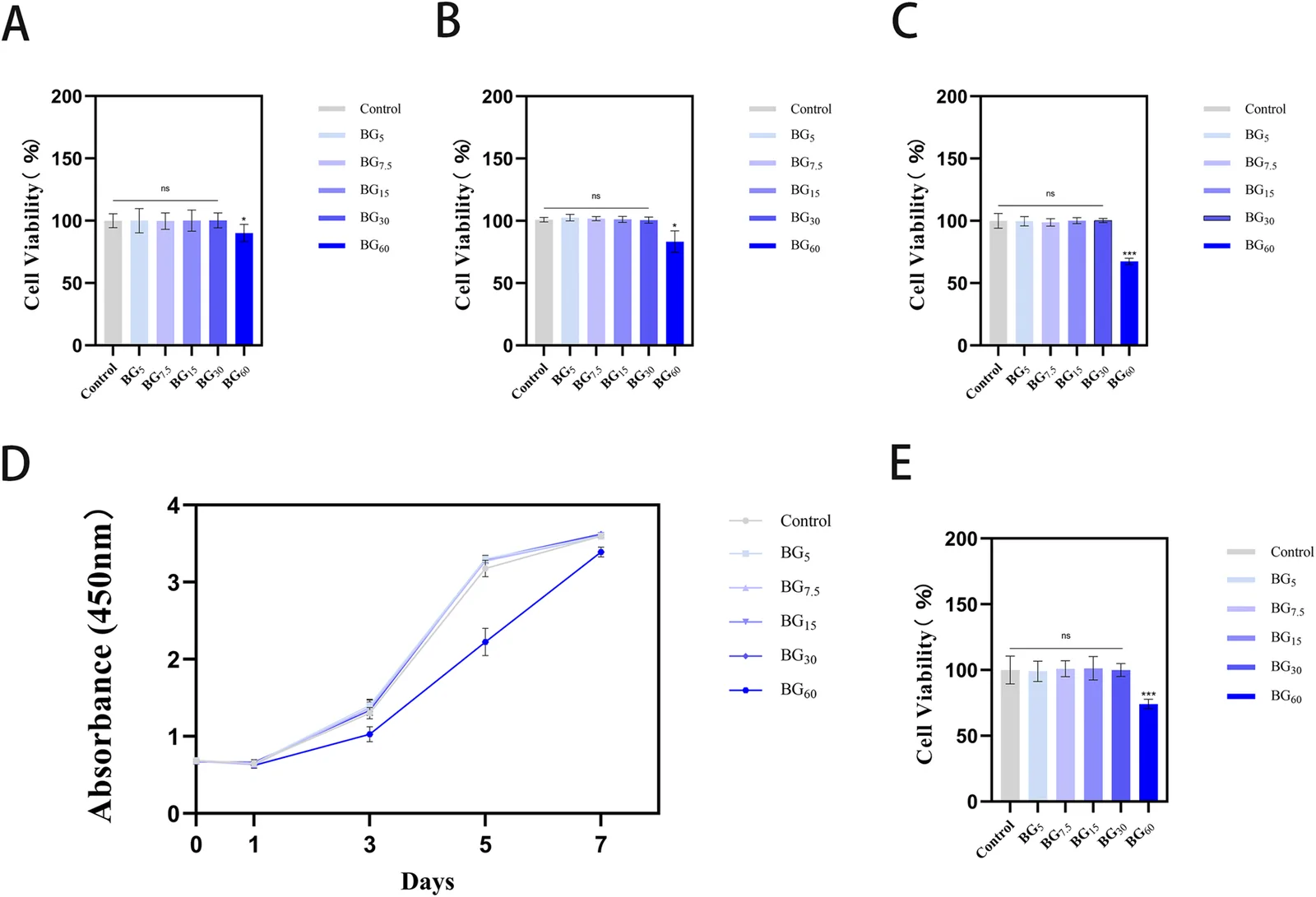
Cytotoxicity of BG. **(A)** 24 h to h-PDLSCs. **(B)** 48 h to h-PDLSCs. **(C)** 72 h to h-PDLSCs. **(D)** 0–7 days to h-PDLSCs. **(E)** 24 h to RAW 264.7 cells. Data are represented as mean ± SD of three independent experiments. *p < 0.05, **p < 0.01, ***p < 0.001.

Similarly, in RAW264.7 cells, acute treatment with 5–30 μM BG for 24 h showed no significant cytotoxicity, with viability comparable to that of the untreated control (*p* > 0.05). However, 60 μM BG notably reduced cell viability (*p* < 0.001; Figure 4E). These results indicate that BG at 5–30 μM is non-cytotoxic to h-PDLSCs and RAW264.7 cells under the tested *in vitro* conditions. However, this does not constitute evidence of general safety or biocompatibility, which would require comprehensive *in vivo* toxicological evaluation.

### 3.4 ROS staining and fluorescence intensity analysis

Fluorescence microscopy revealed that the inflammation group exhibited visibly higher number of DCFH-positive cells compared to all other groups. Although all BG-treated groups displayed more DCFH-positive cells than the normal control, their counts were markedly lower than those in the inflammation group (Figures 5A–F). Quantitative analysis of fluorescence intensity corroborated these observations: the inflammation group displayed the highest intense of green fluorescence among all groups (*p* < 0.001), whereas all BG-treated groups exhibited significantly suppressed fluorescence intensity compared with inflammation group (*p* < 0.001). Notably, the most pronounced ROS scavenging effect was observed in the 7.5–30 μM BG groups, which demonstrated statistically significant reductions in fluorescence intensity relative to both the inflammation group (*p* < 0.001) and the 5 μM BG group (*p* < 0.01) (Figure 5G). Overall, these results highlight the ability of BG to reduce intracellular oxidative activity under periodontitis-like conditions. However, as DCFH-DA measures overall intracellular oxidative activity rather than direct ROS scavenging, the precise mechanism-whether through direct radical quenching or via upregulation of antioxidant enzymes–requires further investigation.

**FIGURE 5:**
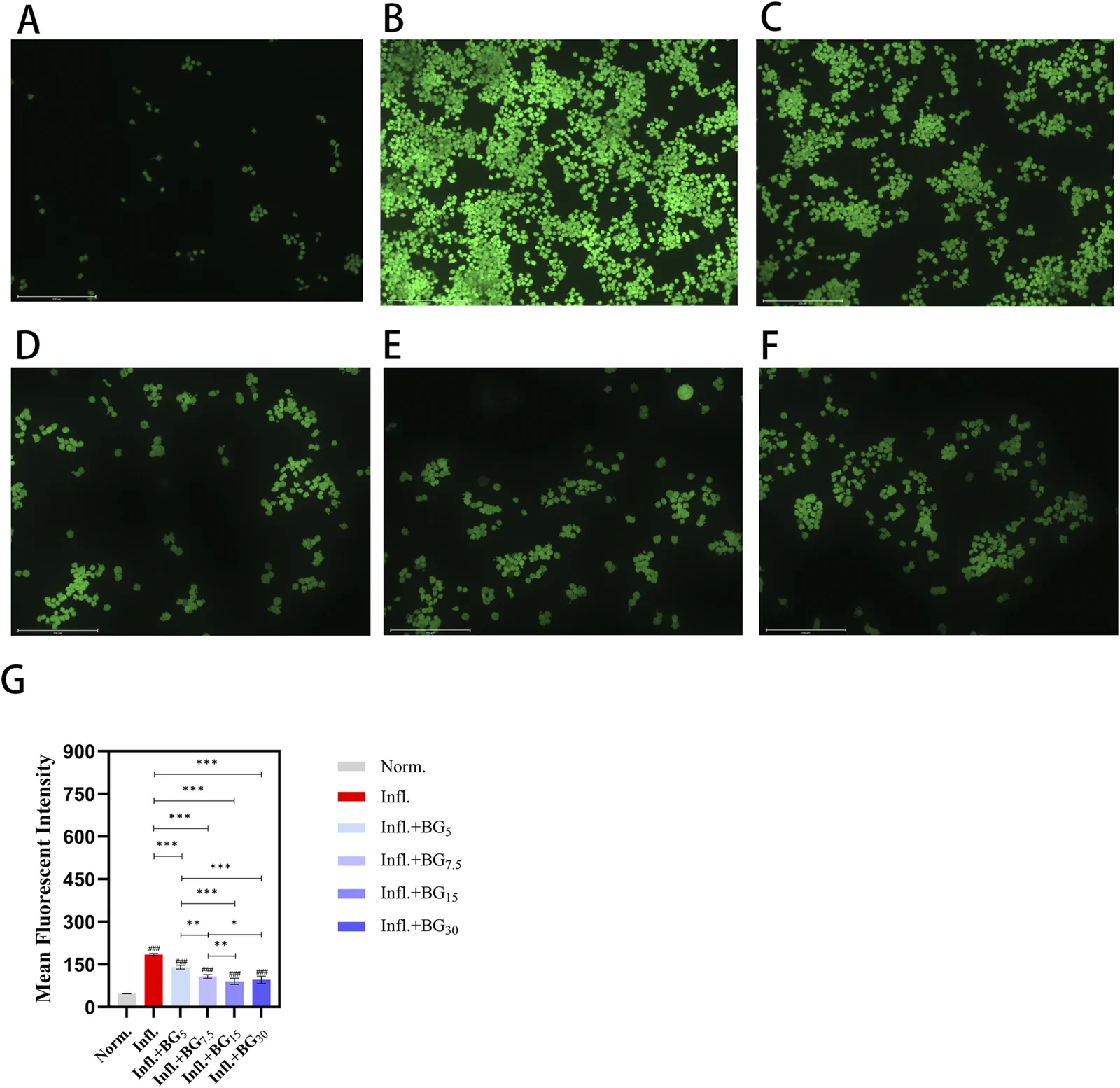
ROS staining and fluorescence intensity analysis. **(A)** Normal group. **(B)** Inflammation group. **(C)** Inflammation +5 μM BG group. **(D)** Inflammation +7.5 μM BG group. **(E)** Inflammation +15 μM BG group. **(F)** Inflammation +30 μM BG group. **(G)** fluorescence intensity analysis. Data are represented as mean ± SD of three independent experiments. *p < 0.05, **p < 0.01, ***p < 0.001. ^#^indicates a statistically significant difference compared with the normal group, ^#^p < 0.05, ^##^p < 0.01, ^###^p < 0.001.

### 3.5 Enzyme-linked immunosorbent assay (Elisa)

As is depicted in Figure 6, the secretion levels of pro-inflammatory cytokines *TNF-*α, *IL-1*β, and *IL-6* were significantly elevated in the inflammation group compared to all other groups (*p* < 0.05). In this inflammatory setting, BG treatment notably suppressed the release of these pro-inflammatory cytokines, with the 15 μM group exhibiting the most marked reduction (*p* < 0.05). In contrast, the 5 μM group did not significantly alter cytokine levels relative to the inflammation group (*p* > 0.05).

**FIGURE 6:**
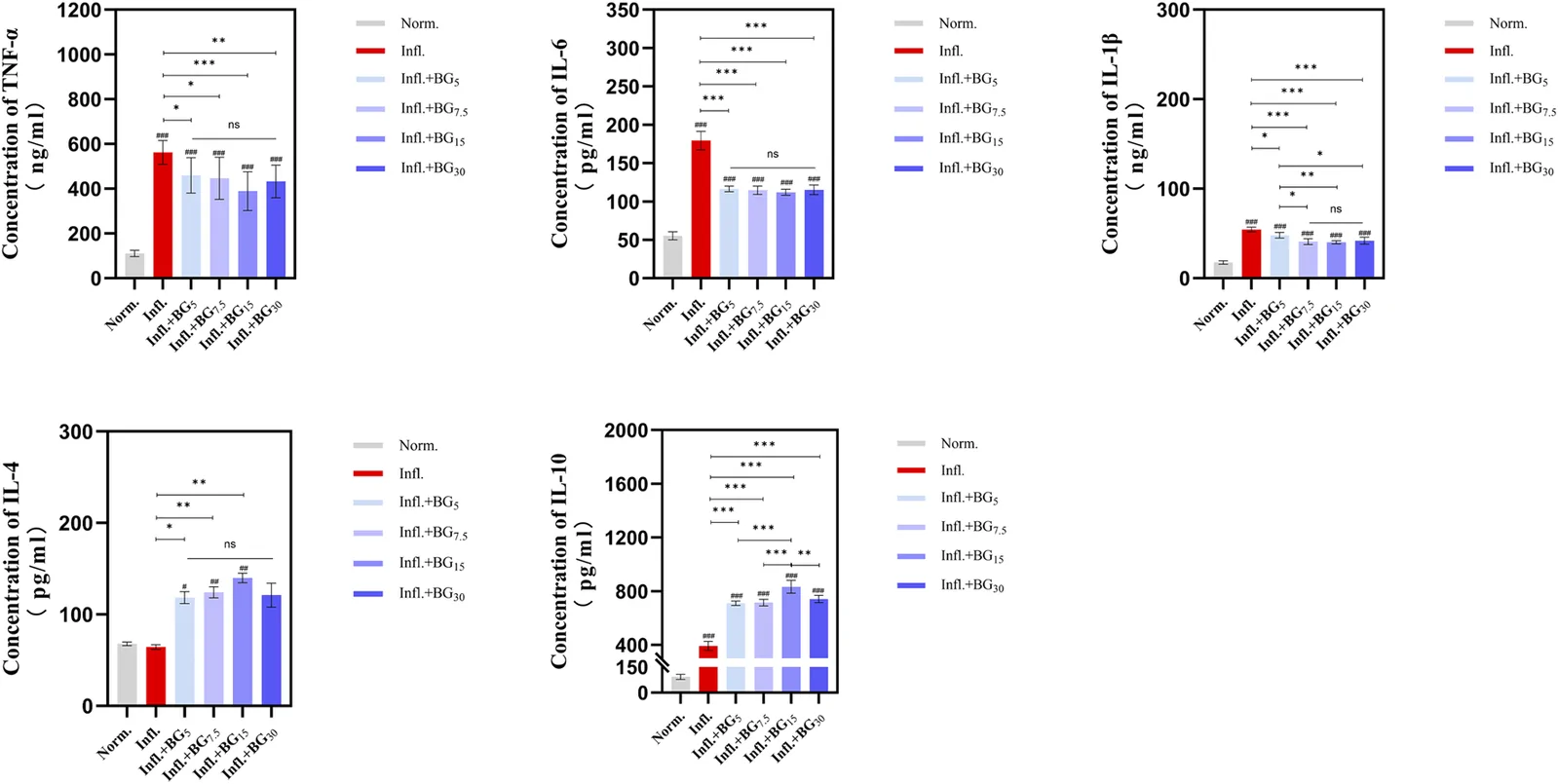
Enzyme-linked immunosorbent assay. Data are represented as mean ± SD of three independent experiments. *p <0.05, **p<0.01, ***p<0.001. ^#^indicates a statistically significant difference compared with the normal group, ^#^p <0.05, ^##^p <0.01, ^###^p <0.001.

Furthermore, BG promoted the expression of anti-inflammatory cytokines. While IL-4 levels remained unchanged in the inflammation group, all BG-treated groups significantly upregulated IL-4 compared to both normal and inflammation groups (*p* < 0.05). IL-10 secretion was elevated in the inflammation group and further enhanced by BG-treated groups, with the 7.5, 15, and 30 μM groups exhibiting higher IL-10 levels than the inflammation group (*p* < 0.05). These results demonstrate that BG not only attenuates pro-inflammatory cytokine release but also actively promotes an anti-inflammatory cytokine profile, supporting its role in reprogramming the immune microenvironment toward a reparative state under periodontitis-like conditions.

### 3.6 Osteogenesis assessment

After 7 days of osteogenic induction, ALP staining and activity assays revealed that all BG treatment groups significantly enhanced both ALP staining intensity (Figures 7A–E) and enzymatic activity (Figure 7K) compared with the normal control (OIM without *P.g*-LPS; *p* < 0.001), with the most pronounced effect observed at 15 μM group compared to other BG treatment groups (*p* < 0.05). Following 21 days of induction, ARS staining and semi-quantitative analysis demonstrated that all BG-treated groups notably promoted calcium disposition formation compared with control group (*p* < 0.01), and 15 μM BG also showed the most pronounced effect compared with all BG treated groups (*p* < 0.001; Figures 7F–J). These results indicate that BG promotes osteogenic differentiation beyond baseline levels in the absence of inflammatory challenge.

**FIGURE 7:**
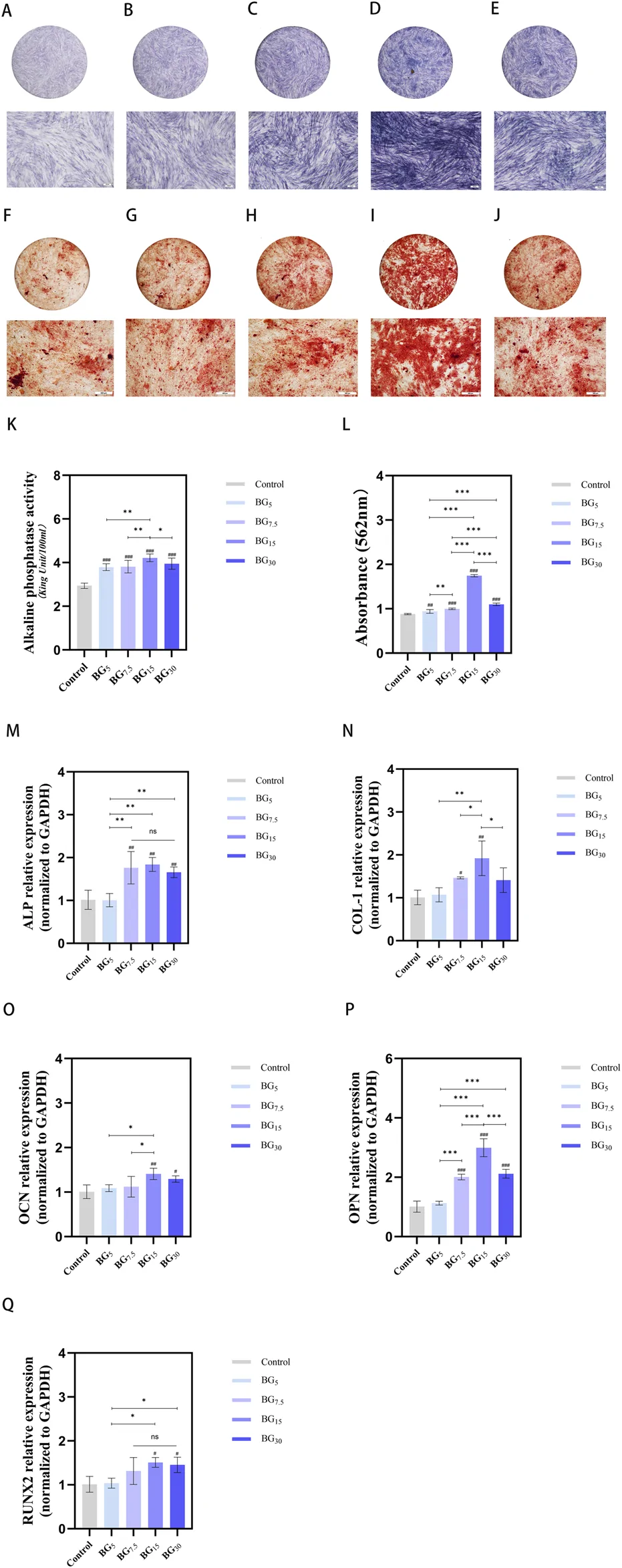
Osteogenesis assessments. **(A–E)** ALP staining. **(F–J)** ARS staining. **(A,F)** Control group. **(B,G)** 5 μM group. **(C,H)** 7.5 μM group. **(D,I)** 15 μM group. **(E,J)** 30 μM group. **(K)** ALP activity analysis. **(L)** ARS semi-quantitation. **(M-Q)** Detection of osteogenic differentiation marker genes. Data are represented as mean ± SD of three independent experiments. *p < 0.05, **p < 0.01, ***p < 0.001. ^#^indicates a statistically significant difference compared with the normal group, ^#^p < 0.05, ^##^p < 0.01, ^###^p < 0.001.

RT-qPCR analysis showed that BG at 7.5–30 μM generally upregulated the mRNA expression of osteogenic markers, including *ALP*, *RUNX2*, *COL-1*, *OCN*, and *OPN*. However, the statistical significance varied among concentrations: the 15 μM group exhibited significant increases in all five genes compared with the control group (*p* < 0.05); the 7.5 μM group significantly upregulated *ALP*, *COL-I*, and *OPN* (*p* < 0.05), but not *RUNX2* or *OCN* (*p* > 0.05); and the 30 μM group significantly elevated *ALP*, *RUNX2*, *OCN* and *OPN* (*p* < 0.05), but not *COL-I* (*p* > 0.05). The 5 μM group showed no significant changes in any of these genes (*p* > 0.05, Figures 7M–Q). These results demonstrate that BG at concentrations of 7.5–30 μM effectively promotes osteogenic differentiation, with 15 μM showing the most pronounced effect. However, no statistically significant differences were observed among the 7.5–30 μM groups for several markers, suggesting a plateau effect within this range. The 5 μM concentration was excluded from subsequent mechanistic analyses because its limited efficacy in the anti-inflammatory (ELISA) and osteogenic gene expression (RT-qPCR) assays. We acknowledge that this exclusion may introduce selection bias, and the lack of efficacy at 5 μM should be interpreted as the limited efficacy threshold within this specific *in vitro* model.

### 3.7 Real-time quantitative polymerase chain reaction analysis and Western blot

As shown in Figures 8A–K, RT-qPCR analysis revealed that the treatment with 7.5–30 μM BG significantly downregulated the mRNA expression of M1 macrophage markers (*iNOS*, *CD86*) and key NF-κB pathway components (*IκBα*, *p65*, *TNF-*α, *IL-6*), compared to the inflammation group. Concurrently, BG treatment upregulated the expression of M2 macrophage markers (*Arg-1*, *CD206*) and antioxidant-related genes (*HO-1*, *CAT*, *SOD*) (*p* < 0.05). The pronounced modulatory effects were observed in the 15 μM group. Notably, within the concentration range of 7.5–30μM, no statistically significant differences were detected in the expression levels of *CD86, CD206, p65, TNF-*α*,* or *IL-6*, suggesting a consistent dose-response plateau. Together, these findings indicate that BG treatment is associated with changes in macrophage polarization-associated markers favoring an M2-like profile, as well as with upregulated antioxidant gene expression and downregulated mRNA levels of NF-κB related inflammatory components. However, as these observations are limited to the mRNA level, they should be interpreted as preliminary evidence rather than definitive proof of macrophage reprogramming or NF-κB pathway inhibition.

**FIGURE 8:**
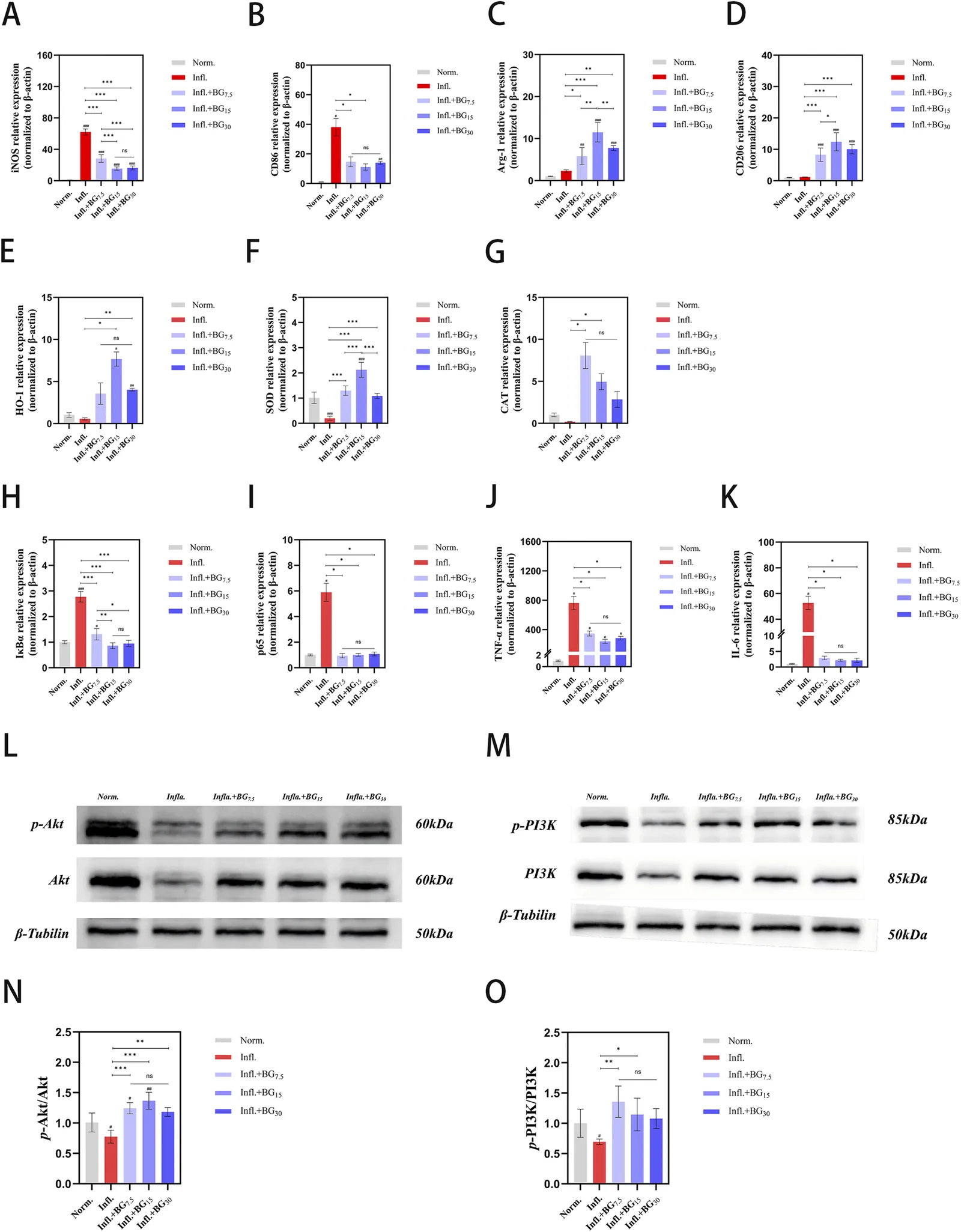
Assessment of molecular markers. **(A–D)** RT-qPCR for M1/M2 macrophage polarization genes. **(E–G)** RT-qPCR for antioxidant genes. **(H–K)** RT-qPCR for NF-κB pathway marker genes. **(L, M)** WB analysis. **(N, O)** Densitometric analysis of p-PI3K/PI3K and p-Akt/Akt ratios. Data are represented as mean ± SD of three independent experiments. *p < 0.05, **p < 0.01, ***p < 0.001. ^#^indicates a statistically significant difference compared with the normal group, ^#^p < 0.05, ^##^p < 0.01, ^###^p < 0.001.

WB analysis showed that the phosphorylation ratios of both p-PI3K/PI3K and p-Akt/Akt were significantly decreased in the inflammation group compared to the normal group (*p* < 0.05). Treatment with BG restored these ratios under periodontitis-mimicking conditions. Specifically, for p-PI3K/PI3K, the 7.5 and 15 μM BG groups exhibited significantly higher ratios than the inflammation group (*p* < 0.05), whereas 30 μM group did not show a statistically significant difference compared to the inflammation group (*p* > 0.05). For p-Akt/Akt, all three BG treatment groups showed significant increases compared to the inflammation group (*p* < 0.05; Figures 8L–Q). Notably, no statistically significant differences were observed among the three BG concentration groups for either ratio (*p* > 0.05), suggesting a plateau effect within this concentration range. These results indicate that BG treatment is associated with increased phosphorylation levels of PI3K and Akt in the periodontitis-mimicking conditions, suggesting a potential involvement of this pathway in the observed pro-osteogenic and cytoprotective effects.

### 3.8 Osteoimmunomodulation analysis

ALP staining/activity and ARS staining/semi-quantification showed that CM from inflammation group markedly inhibited osteogenic differentiation of h-PDLSCs, as demonstrated by minimal ALP staining intensity, the lowest ALP activity, and scarce mineralized nodule formation (*p* < 0.05). In contrast, CM derived from all BG-treated macrophage groups significantly attenuated this inhibitory effect, resulting in enhanced staining, elevated ALP activity, and increased matrix mineralization relative to the inflammation group (*p* < 0.05; Figures 9A–L). Nevertheless, the ALP activity and mineralization levels in these groups remained lower than those in the normal control group (CM from untreated macrophage without *P.g*-LPS), indicating that the conditioned medium from BG-treated macrophages only partially rescued the osteogenic capacity of h-PDLSCs.

**FIGURE 9:**
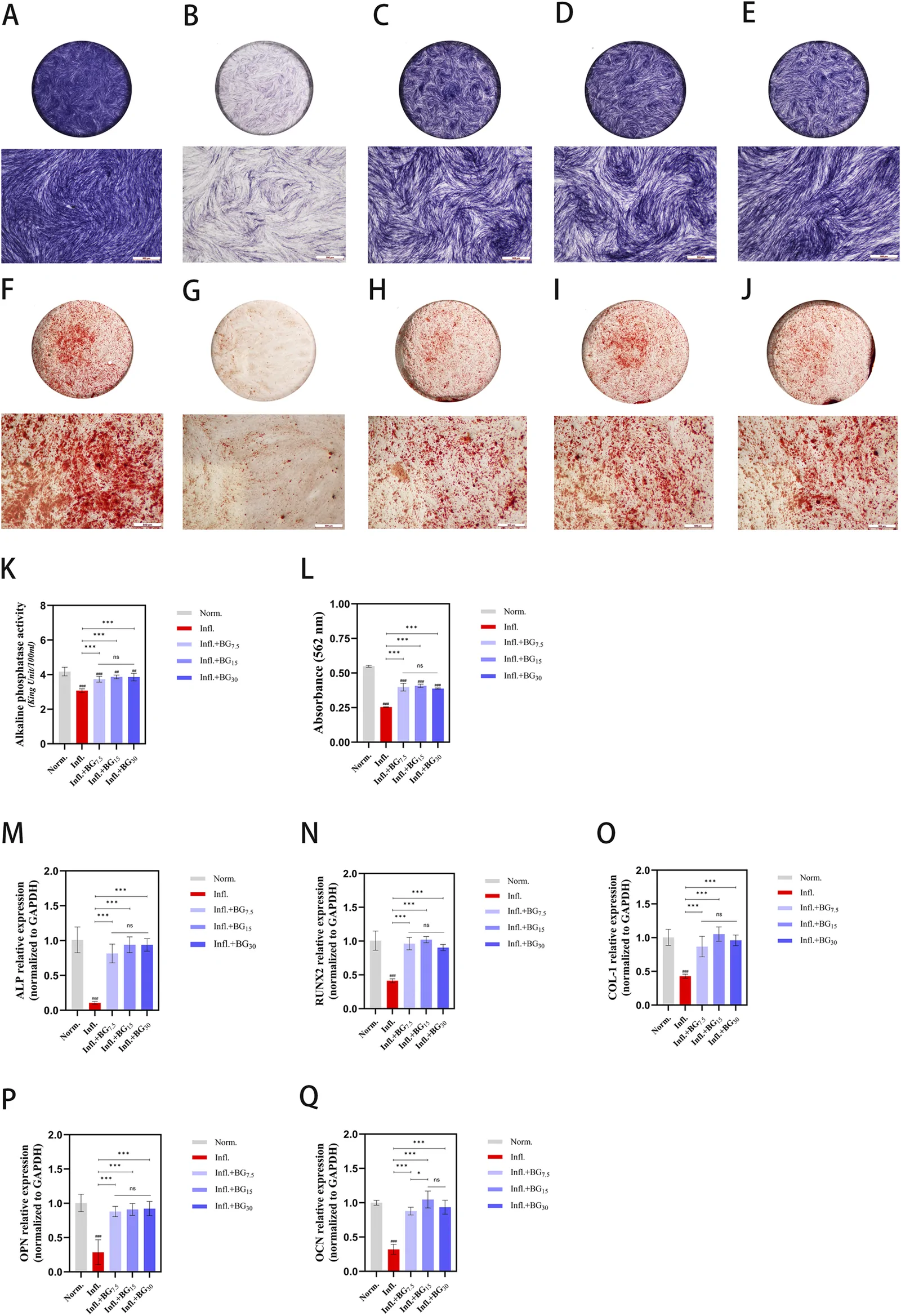
Osteoimmunomodulation analysis. **(A–E)** ALP staining. **(F–J)** ARS staining. **(A, F)** Normal group. **(B, G)** Inflammation group. **(C, H)** Inflammation +7.5 μM BG group. **(D, I)** Inflammation +15 μM BG group. **(E, J)** Inflammation +30 μM BG group. **(K)** ALP activity analysis. **(L)** ARS semi-quantitation. **(M–Q)** Detection of osteogenic differentiation marker genes. Data are represented as mean ± SD of three independent experiments. *p < 0.05, **p < 0.01, ***p < 0.001. ^#^p < 0.05, ^##^p < 0.01, ^###^p < 0.001.

Consistent with these phenotypic observations, RT-qPCR analysis revealed that the expression of osteogenic marker genes (*ALP, RUNX2, COL-1, OCN, OPN*) was most suppressed in the inflammatory group (*p* < 0.05). All BG-treated CM groups significantly upregulated these markers relative to the inflammatory group (*p* < 0.05), with no concentration-dependent variations observed across different BG doses (Figures 9M–Q). These findings suggest that BG-treated macrophages release soluble factors that may contribute to an osteogenic microenvironment. However, as the specific factors responsible for this effect have not been identified, this observation should be interpreted as preliminary evidence of paracrine crosstalk between macrophages and h-PDLSCs rather than a defined mechanistic pathway, and the possibility of residual BG in the conditioned medium directly affecting h-PDLSCs cannot be excluded. In addition, potential interference from BG as a pan-assay interference compound (PAINS) was considered; however, the specific, concentration-associated responses observed argue against a non-specific effect.

## 4 Discussion

Periodontitis is a multifactorial chronic disease hallmarked by persistent inflammation, unbated oxidative stress, and progressive alveolar bone resorption. Current therapeutic regimes, which predominantly target single pathological aspects, are inherently insufficient to address the complex interplay of pathogenic drivers, often resulting in suboptimal clinical outcomes. The main contributions of this study are threefold. We systematically evaluated BG’s effects on inflammation-related cytokine production, oxidative activity, and osteogenic marker expression in a *P.g*-LPS induced periodontitis-mimicking model. We also observed that conditioned medium form BG-treated macrophages were sufficient to partially restore the osteogenic capacity of h-PDLSCs, suggesting a paracrine component in BG’s action. Finally, we integrated network analysis with *in vitro* experiments to generate testable hypotheses regarding candidate pathways, including PI3K/Akt and NF-κB. Together, these observations suggest that BG exhibits multimodal activities relevant to the pathological features of periodontitis.

The dysregulated host immune response, particularly the imbalance of M1/M2 macrophage polarization with a predominance of the pro-inflammatory M1 phenotype, is closely correlated with disease severity and refractory progression, as sustained activation of M1-polarized macrophages leads to excessive release of proinflammatory cytokines and ROS, creating a self-perpetuating cycle of oxidative stress and tissue destruction that disrupts periodontal homeostasis and impairs the osteogenic capacity of h-PDLSCs (Peng et al., 2024; Ruytinx et al., 2018; Kanzaki et al., 2017). In our *in vitro P.*g-LPS induced periodontitis-mimicking model, the elevated pro-inflammatory cytokines, ROS accumulation, M1 phenotypic dominance, and suppressed antioxidant defense that collectively compromised h-PDLSCs osteogenic capacity were associated with BG treatment, which corresponded to reduced mRNA expression of M1 macrophage markers and NF-κB components, along with increased mRNA expression of M2 macrophage markers and antioxidant related genes. These findings are in line with previous reports suggesting that BG may modulate macrophage polarization state (Liao et al., 2025) and influence inflammatory responses (Zheng et al., 2025), as well as with studies indicating that BG is associated with enhanced activities of catalase and superoxide dismutase in other experimental systems (Chen et al., 2026)^.^ Regarding the oxidative stress measurements, it should be noted that the DCFH-DA assay used in this study reflects overall intracellular oxidative activity rather than direct ROS scavenging. Therefore, whether BG acts through direct radical quenching, indirect upregulation of antioxidant enzymes, or a combination of both remains to be determined. Notably, although *CAT* expression at 15 μM was slightly lower than at 7.5 μM with no statistical difference, likely due to context-dependent regulation of Nrf2 targets, *HO-1* and *SOD* peaked at 15 μM, and this gene-specific response requires further investigation. It is important to acknowledge that our assessment of macrophage polarization markers was limited to the mRNA level, and protein-level validation (e.g., via flow cytometry or immunofluorescence) was not performed. Thus, our RT-qPCR data indicate an association between BG treatment and changes in macrophage polarization related markers, rather than definitive evidence of macrophage reprogramming.

Beyond its anti-inflammatory and antioxidative actions, BG exhibited osteogenic effect that operated through distinct mechanisms depending on the microenvironment. In the absence of inflammatory challenge, BG directly promoted the osteogenic differentiation of h-PDLSCs to levels exceeding those of the osteogenic control, as evidenced by increased ALP activity, mineralization, and upregulation of osteogenic markers. Previous studies have reported that BG enhances ALP activity and BMP-2 expression in osteoblasts via p38 and ERK-dependent signaling pathways, suppresses NF-κB and JNK signaling, and promotes bone marrow mesenchymal stem cells osteogenic differentiation both *in vitro* and *in vivo* (Chen et al., 2019; Shen et al., 2023; Xiao et al., 2015). Notably, while ALP staining and ARS semi-quantification showed that even 5 μM BG enhanced enzymatic activity and mineralization, the corresponding mRNA levels of osteogenic genes at this concentration didi not reach statistical significance. This dissociation between functional activity and transcriptional response is not uncommon in osteogenesis research; it may reflect post-transcriptional regulation, differences in the sensitivity of enzymatic *versus* genomic readouts, or the fact that ALP activity and mineralization represent cumulative functional outcomes that integrate multiple regulatory inputs beyond transcriptional induction alone (Benz et al., 2013). Under inflammatory conditions, however, BG appeared to shift its mode of action. Instead of acting directly on h-PDLSCs, BG-treated macrophages released soluble factors that partially restored the osteogenic capacity of h-PDLSCs, as demonstrated by the conditioned medium experiments. However, the osteogenic levels achieved remained below those of the normal control, indicating a partial rescue rather than a complete reversal of inflammation induced suppression. Moreover, it should be acknowledged that the conditioned medium experiments, while suggestive of macrophage-mediated effects, do not completely exclude the possibility that residual BG carried over into the conditioned medium or pharmacologically inhibited macrophages would be required to definitively establish the indirect mechanism. This osteoimmunomodulatory observation suggest that a paracrine component may involved in BG’s action, as the conditioned medium from BG-treated macrophages partially rescued inflammation-impaired osteogenic differentiation of h-PDLSCs. This effect is distinct from the direct osteogenic effect of BG. Additionally, while the conditioned medium experiments suggested that BG-treated macrophages release soluble factors capable of influence h-PDLSCs osteogenesis, we did not identify the specific factors responsible for this effect. Cytokine profile revealed changes in secreted IL-4, IL-10, TNF-α, IL-1β, and IL-6 levels, but neutralization experiments or mediator-specific loss-of-function approaches were not performed. Therefore, the observed paracrine effect should be interpreted at the population level rather than attributed to any specific molecular mediator.

Mechanistically, the crosstalk between pro-inflammatory and pro-regenerative signaling pathways critically dictates the fate of h-PDLSCs (Hu et al., 2023). Persistent activation of the NF-κB pathway by LPS drives sustained transcription of inflammatory mediators while suppressing osteogenic programs (Barnabei et al., 2021; Tang et al., 2024). Conversely, the PI3K/Akt pathway promotes osteoblast differentiation and maintains skeletal homeostasis but is markedly suppressed in the inflammatory niche (Zhao et al., 2020; Han et al., 2024). In our study, network analysis predicted PI3K/Akt as a top-ranked candidate pathway for BG. Western blot analysis showed that BG treatment was associated with increased phosphorylation ratios of p-PI3K/PI3K and p-Akt/Akt, which were reduced upon *P.g*.-LPS stimulation. Notably, the 30 μM group did not show a statistically significant restoration of p-PI3K/PI3K compared to the inflammation group, while p-Akt/Akt was significantly restored at all three concentrations. This discrepancy may reflect differential sensitivity of PI3K and Akt phosphorylation to BG, or the involvement of feedback regulatory mechanisms, and warrants further investigation. However, as we did not employ pharmacological inhibitors or activators, these findings are correlational and do not establish a causal relationship. Therefore, we conclude that the PI3K/Akt pathway is a candidate pathway associated with BG’s effects, rather than a definitively demonstrated mediator. Furthermore, NF-κB pathway components were assessed only at the mRNA level, and protein-level validation (e.g., *IκBα* degradation or *p65* nuclear translocation by immunofluorescence or Western blot) was not performed. Therefore, definitive conclusions regarding NF-κB pathway inhibition require further functional studies. Our data indicate a correlational relationship of NF-κB-related transcription. Therefore, while our data identify PI3K/Akt and NF-κB as candidate pathways associated with the multimodal effects of BG, definitive mechanistic conclusions, particularly regarding pathway activation or inhibition, require further investigation of function approaches.

Despite the promising multi-target profile of BG, several limitations should be acknowledged. First, the *P.g*-LPS stimulated RAW264.7 macrophage and h-PDLSCs co-culture model represents a simplified *in vitro* surrogate for the complex periodontal microenvironment. This model does not recapitulate the full polymicrobial, three-dimensional, and dynamically remodeling nature of clinical periodontitis. Therefore, our findings should be considered as proof-of-concept evidence in a periodontitis-like inflammatory setting, and *in vitro* validation in animal periodontitis model is required before translational conclusions can be drawn. Second, the mechanistic understanding of BG-mediated macrophage polarization, anti-inflammatory effects, and antioxidant responses remains preliminary, as most assessments were limited to the mRNA level. Third, it should be noted that our study revealed a correlational relationship between BG treatment and increased PI3K/Akt phosphorylation and decreased mRNA of NF-κB pathway components. As we did not employ pharmacological inhibitors, genetic knockdown approaches, these findings do not establish a causal role for PI3K/Akt or NF-κB in mediating the observed effects. Therefore, PI3K/Akt and NF-κB pathways should be considered a candidate pathway associated with BG’s treatment, and future studies using pathway-specific interventions are warranted to test this hypothesis. Fourth, as a small-molecule agent, BG lacks sufficient local retention at periodontal lesions, highlighting the need for advanced delivery systems such as hydrogels or scaffolds to achieve sustained and targeted therapy. At last, the absence of *in vivo* data is a major limitation of this study. Future work using animal periodontitis models (e.g., ligature-induced or *P.g*-infected models) will be essential to assess the therapeutic efficacy and safety of BG in a physiologically complex environment.

## 5 Conclusion

In summary, this *in vitro* study suggests that BG possesses multimodal activities relevant to periodontitis. By integrating network analysis with experimental validation, we observed that BG treatment was associated with reduced inflammatory responses, decreased intracellular oxidative activity, increased expression of M2-associated and oxidant-related genes, enhances osteogenic differentiation, and increased phosphorylation ratios of PI3K and Akt. These coordinated *in vitro* activities suggest BG as a candidate for further *in vivo* evaluation for periodontitis. These findings indicate that BG exhibits multimodal features that warrant further *in vivo* evaluation as a potential candidate for periodontal therapy. Future studies using pathway-specific inhibitors and animal models are essential to test the candidate mechanisms identified in this work (Figure 10).

**FIGURE 10:**
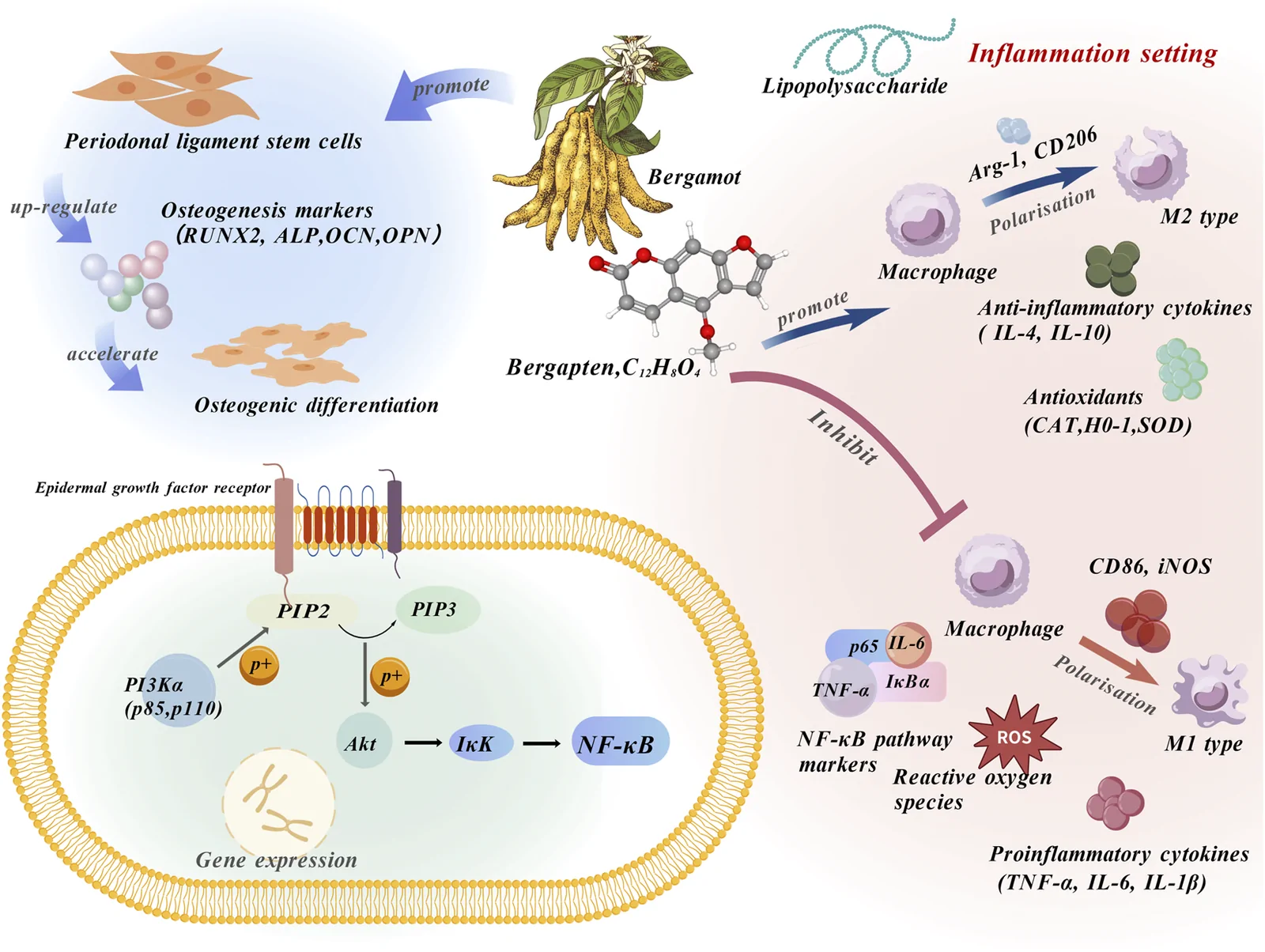
Diagram of discussion.

## Data Availability

The datasets generated and/or analyzed during the current study are not publicly available due to the ethical and privacy considerations but are available from the corresponding author on reasonable request.
